# Spontaneous intramural hematoma of the small bowel in anticoagulated patients—Insights into management: A case report

**DOI:** 10.1016/j.radcr.2025.06.079

**Published:** 2025-07-28

**Authors:** Ali Hajihashemi, Hadi Khanifar, Mahsa Geravandi

**Affiliations:** a Department of Radiology, School of Medicine, Isfahan University of Medical Sciences, Isfahan, Iran; b Department of Internal Medicine, Shahre-kord University of Medical Sciences, Shahre-kord, Iran

**Keywords:** Small bowel intramural hematoma, Anticoagulation therapy, Warfarin complications, Abdominal pain, Case report

## Abstract

Intramural hematoma of the small bowel is a rare but potentially life-threatening complication of anticoagulation therapy, most commonly associated with warfarin use. This report presents two cases highlighting the clinical presentation and management of this condition. The first case involved a 72-year-old male with a history of heart valve replacement who presented with severe abdominal pain, nausea, and vomiting; imaging revealed a jejunal intramural hematoma and a significantly elevated INR. The second case involved a 51-year-old female with atrial fibrillation who experienced acute abdominal pain and partial bowel obstruction; CT imaging showed an intramural hematoma in the ileum with associated hemoperitoneum. Both patients were treated conservatively through cessation of warfarin, administration of vitamin K, and transfusion of fresh-frozen plasma, with one requiring surgical exploration but no bowel resection. Full recovery was achieved in both cases, and patients were safely transitioned to direct oral anticoagulants with no recurrence. These cases underscore the need for prompt diagnosis and multidisciplinary management in anticoagulated patients presenting with abdominal symptoms to prevent serious complications and improve outcomes.

## Background

Abdominal pain is one of the most common complaints presented to emergency departments, ranging from mild to severe conditions [1]. Accurate diagnosis requires careful assessment of pain location, associated symptoms, medical history, and imaging studies [2]. While common diagnoses include gastroenteritis, irritable bowel syndrome, and urologic disorders, less frequent diagnoses such as intramural hematoma in the small intestine should not be missed, especially in patients on anticoagulation therapy. Small Bowel Intramural Hematoma (SBIMH), most commonly linked to anticoagulant therapy, occurs in approximately 1 in 2500 patients on warfarin annually [3]. This condition, though rare, represents a critical differential diagnosis due to its potentially severe complications and serious clinical challenges [3,4]. However, the majority of these cases are small and can be managed non-invasively if they are diagnosed in time. Therefore, recognizing SBIMH as a potential etiology in patients with abdominal pain is critical to optimizing outcomes [5]. Clinical presentation includes abdominal pain, nausea, vomiting, and occasionally, intestinal obstruction or gastrointestinal bleeding. Prompt diagnosis is essential and Computed tomography (CT) is the imaging modality most commonly used in diagnosis. CT findings include wall thickening, luminal narrowing, intramural hyper-density, and obstruction of the intestine. The occurrence of hematoma is most frequently observed in the jejunum (69%), followed by the ileum (38%), and the duodenum (23%), with some cases involving multiple contiguous segments, which accounts for the cumulative percentages exceeding 100%. Conservative management, including discontinuation or reversal of anticoagulation and supportive care, is typically effective. Surgery is reserved for complications such as bowel obstruction or perforation [6,7].

## Case 1

A 72-year-old Iranian male, a retired accountant with a body mass index (BMI) of 28.5 kg/m² (classified as overweight), presented to the Emergency Department complaining of abdominal pain. He lived independently with his spouse. The patient reported a gradual onset of abdominal pain over two days, localized to the periumbilical and left lower quadrant regions. The pain was described as dull, non-radiating, and associated with frequent (3-4 times) episodes of vomiting and nausea. He walked unaided into the Emergency Department for evaluation.

The patient had a history of mechanical heart valve replacement and had been on long-term warfarin therapy (5 mg once daily) for the past five years. His medical history also included hypertension, managed with amlodipine (5 mg once daily), and stage III chronic kidney disease (CKD). He denied any recent changes to his medication regimen, over-the-counter drug use, or use of herbal supplements. There was no history of gastrointestinal disease, bowel habit changes, or prior abdominal surgeries. Given his impaired renal function, reduced clearance of medications may have increased his sensitivity to warfarin, contributing to a supratherapeutic INR and elevated bleeding risk. Additionally, he reported no family history of bleeding disorders or significant gastrointestinal diseases. He was a former smoker with a 25-pack-year history but had quit smoking five years prior to presentation.

On physical examination, the patient was hemodynamically stable, with a blood pressure of 135/80 mmHg and a regular heart rate of 80 beats per minute. No pallor, jaundice, or peripheral edema was observed.

During abdominal examination, mild tenderness was noted in the periumbilical region, without guarding or rebound tenderness. There were no palpable masses or organomegaly, and bowel sounds were present but hypoactive. Other physical examination findings were unremarkable (Table 1).

**Table 1:** Summary of laboratory findings.

Test	Result	Baseline/Reference range	Interpretation
Hemoglobin	12 g/dL	13.0 g/dL (baseline)	Mild anemia
Red blood cell count (RBC)	5.2 × 10⁶/µL	4.8 × 10⁶/µL (baseline)	Slightly elevated
White blood cell count (WBC)	11 × 10³/µL	8 × 10³/µL (baseline)	Mild leukocytosis, suggestive of stress/inflammation
Platelets	313 × 10³/µL	320 × 10³/µL (baseline)	Stable and within normal limits
Creatinine	2.7 mg/dL	1.5 mg/dL (baseline)	Elevated, consistent with stage III CKD
Prothrombin time (PT)	79 s	14-16 s	Significantly prolonged
International normalized ratio (INR)	6.5	2-3 (therapeutic range)	Markedly elevated, high risk of bleeding
Partial thromboplastin time (PTT)	32 s	30 s (baseline)	Within normal limits

Note: Urinalysis and serum electrolyte levels were within normal limits, with no significant deviations from baseline values.

Differential diagnoses considered included mechanical bowel obstruction, gastrointestinal perforation, and ischemic bowel disease.

**Fig. 1:**
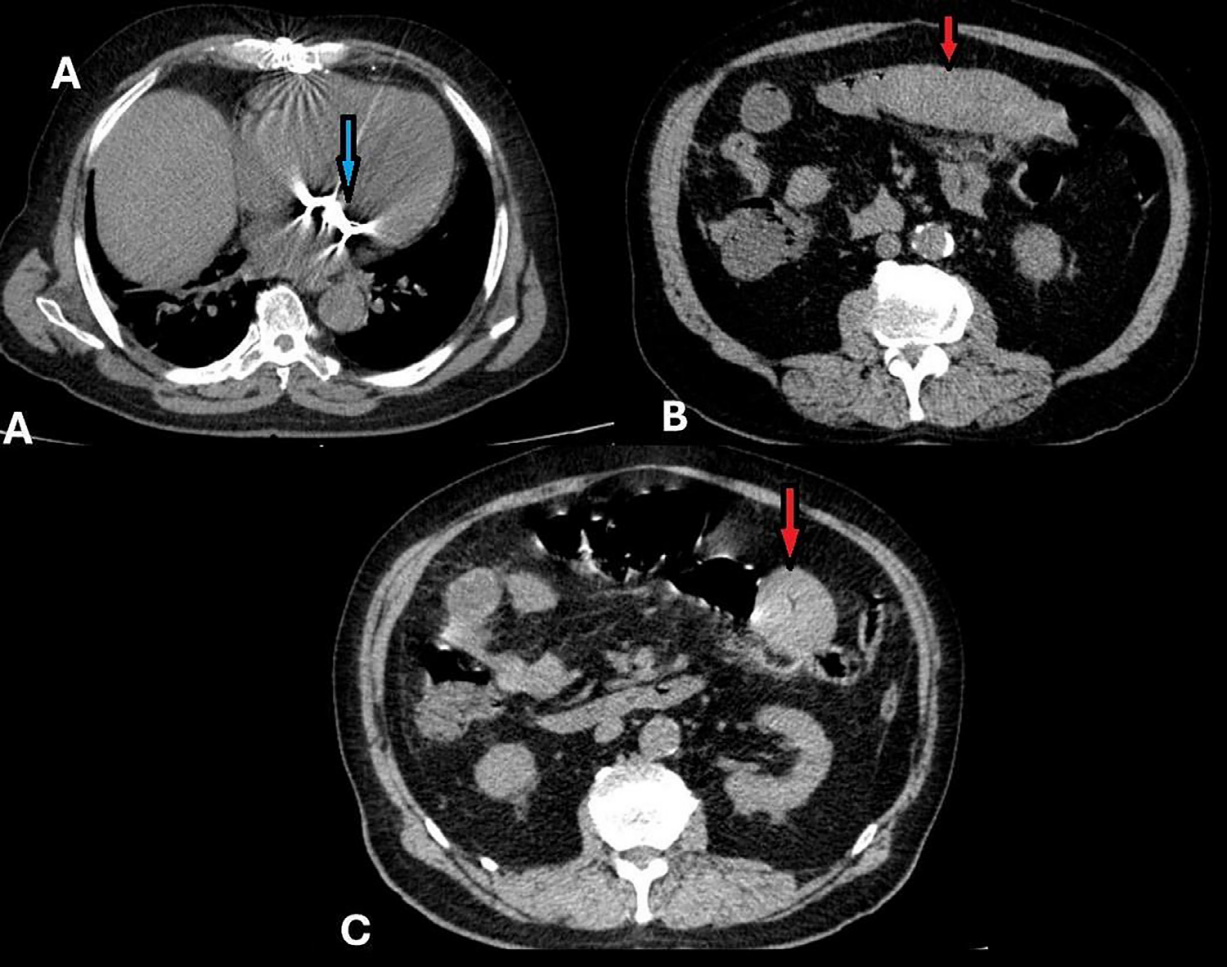
Nonenhanced Abdominopelvic computed tomography (CT) images. (A) Evidence of prior sternotomy and mitral valve replacement is visible (Blue arrow). (B and C) The jejunal loop shows hyperdense long-segment circumferential wall thickening, with adjacent mesenteric haziness and fluid (red arrows).

**Fig. 2:**
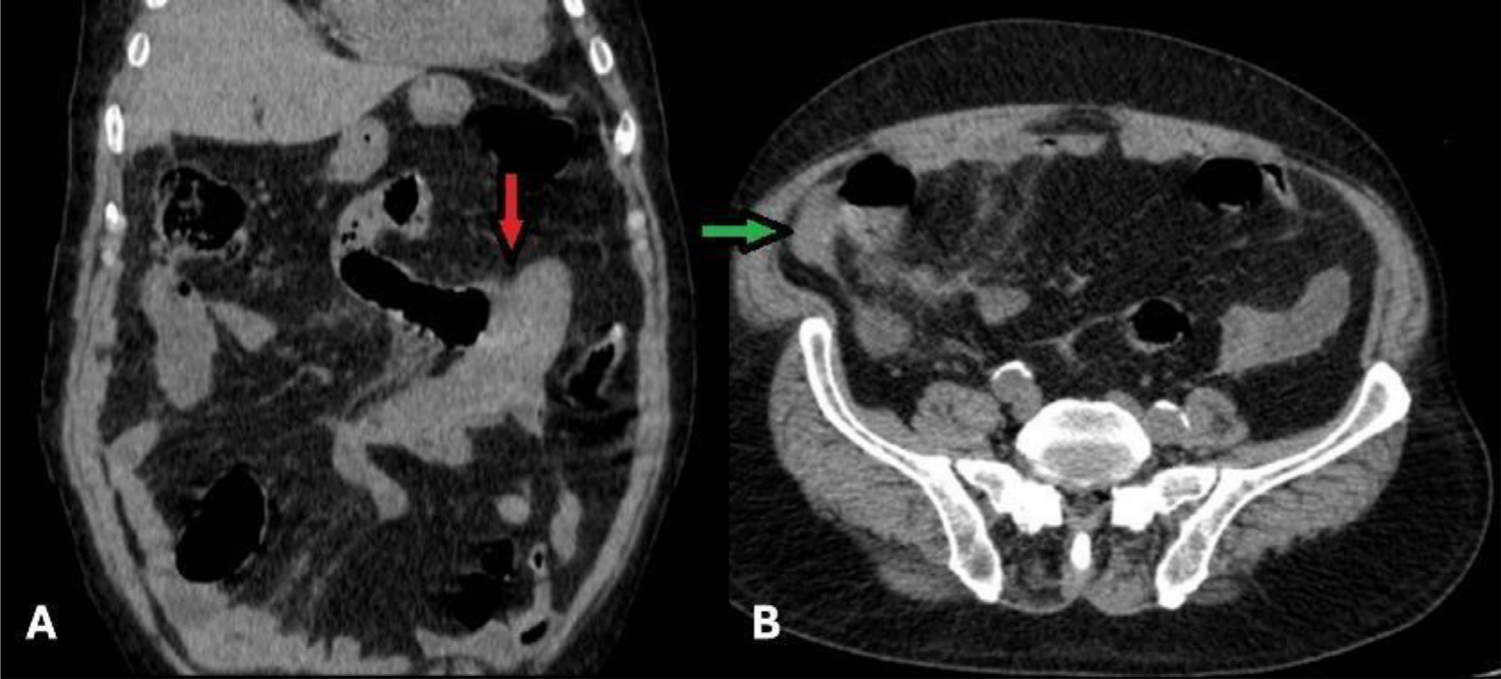
Nonenhanced Abdominopelvic computed tomography (CT) images. (A) Coronal view demonstrates a jejunal loop with circumferential long-segment wall thickening, appearing hyperdense, consistent with an intramural hematoma (IMH). (B) Hyper-attenuated free fluid in the right para-colic gutter, indicative of mild hemoperitoneum, is observed (green arrow).

An initial abdominal and pelvic ultrasound revealed thickened bowel loops as the only notable finding. (Not shown). Consequently, a CT scan was recommended for further evaluation. Due to the patient’s chronic kidney disease, a non-enhanced abdominopelvic CT scan was performed to avoid contrast-induced nephropathy. The scan revealed a hyper-attenuated, long-segment circumferential thickening of the jejunal bowel wall, consistent with an intramural hematoma. The involved jejunal segment demonstrated symmetrical wall thickening extending over a considerable length, indicative of extensive bowel wall involvement. Adjacent mesenteric haziness and minimal fluid accumulation were noted, suggesting mild surrounding inflammation but no evidence of perforation or pneumoperitoneum (Fig. 1). The hyperdense appearance of the thickened bowel wall is characteristic of acute hemorrhage within the wall layers. Coronal views further confirmed the circumferential and long-segment nature of the jejunal involvement, with hyperdense free fluid detected in the right paracolic gutter, consistent with mild hemoperitoneum (Fig. 2).The diagnosis of intramural bowel hematoma was confirmed based on imaging findings and the patient's history of anticoagulation therapy.

Warfarin therapy was immediately discontinued. Fresh-frozen plasma was administered, and vitamin K therapy was initiated to correct the elevated INR prior to surgery.

Supportive care included bowel rest, intravenous hydration, and symptomatic management with analgesics and anti-emetics as needed.

**Fig. 3:**
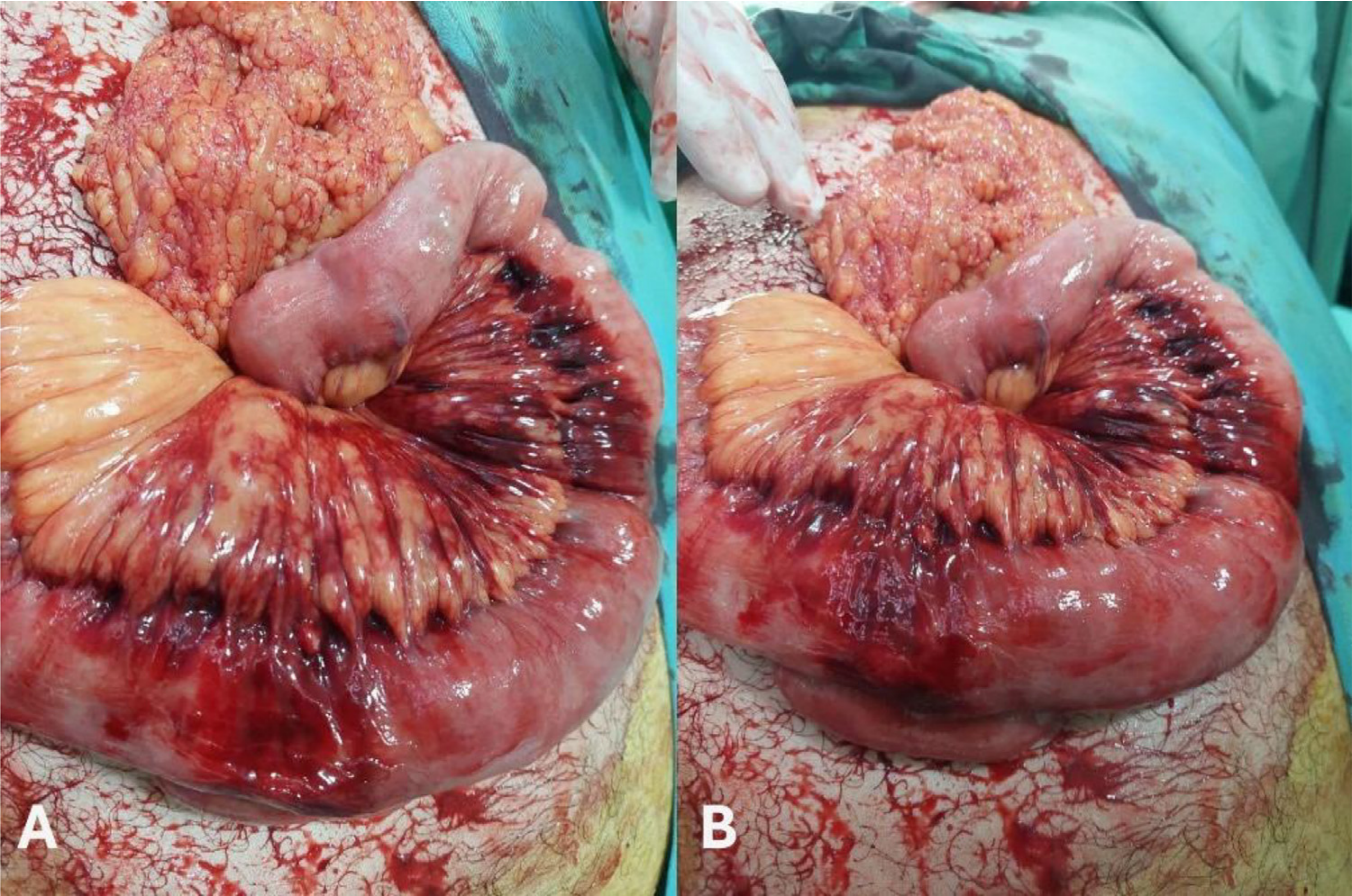
Intraoperative image (A and B) shows localized small intestinal intramural hemorrhage without evidence of active bleeding or ischemia.

Under general anesthesia, laparotomy was performed due to clinical suspicion of intraperitoneal bleeding despite the preoperative imaging diagnosis of intramural hematoma (Fig. 3). Intraoperative findings confirmed localized small intestinal intramural hemorrhage without evidence of active bleeding or ischemia. Therefore, no bowel resection or further surgical intervention was necessary, and the abdomen was closed following thorough inspection.

The patient showed significant symptomatic improvement within 48 hours.

Follow-up sonography performed three weeks after discharge demonstrated complete resolution of the intramural hematoma. Anticoagulation therapy was resumed, with a transition to direct oral anticoagulants (DOACs) to minimize the risk of recurrence. The transition was uneventful, and no complications or adverse events were noted during hospitalization or follow-up

## Case 2

A 51-year-old Iranian female, a retired teacher with a body mass index (BMI) of 26.3 kg/m² (classified as overweight), presented to the Emergency Department with a history of atrial fibrillation, which had been managed with warfarin (5 mg/day) for the past eight months. She reported acute, severe abdominal pain that had persisted for the past 12 hours. The pain was diffuse but more pronounced in the right lower quadrant. Additionally, she noted mild bloating and constipation over the previous week. She denied experiencing nausea, vomiting, or rectal bleeding. The patient was able to walk independently into the Emergency Department.

She had a medical history of atrial fibrillation and had been taking warfarin (5 mg daily) regularly and consistently over the past eight months, with no missed doses or recent medication changes. Her comorbidities included type 2 diabetes mellitus, controlled with metformin (500 mg twice daily), and osteoarthritis, for which she reported occasional use of nonsteroidal anti-inflammatory drugs (NSAIDs). She acknowledged taking several doses of NSAIDs during the week prior to symptom onset, although she could not recall the exact timing of the last dose relative to the onset of abdominal pain. This intermittent NSAID use may have potentiated the anticoagulant effect of warfarin by impairing gastrointestinal mucosal defenses and platelet function. The combination of anticoagulation and recent NSAID exposure likely increased her overall bleeding risk and contributed to the development of the intramural hematoma.

She had no history of prior surgical interventions and no significant family history of bleeding disorders or gastrointestinal diseases. The patient was a non-smoker.

On general examination, she was hemodynamically stable, with a blood pressure of 125/78 mmHg, a heart rate of 72 bpm (irregular), a respiratory rate of 16 breaths per minute, and a temperature of 36.8°C. Mild bruising was observed on her arms and legs, likely associated with anticoagulation therapy. On abdominal examination, there was diffuse tenderness, particularly in the right lower quadrant, but without rebound tenderness or guarding. Bowel sounds were present but hypoactive (Table 2).

**Table 2:** Laboratory findings on admission.

Parameter	Value on admission	Baseline value	Reference range	Interpretation
Hemoglobin	13 g/dL	12.1 g/dL	12-16 g/dL (female)	Slight increase from baseline; within normal limits
RBC count	4.7 × 10⁶/µL	4.5 × 10⁶/µL	4.2-5.4 × 10⁶/µL	Normal
WBC count	9 × 10³/µL	8 × 10³/µL	4-10 × 10³/µL	Mildly elevated; possible stress or inflammation
Platelet count	386 × 10³/µL	250 × 10³/µL	150-400 × 10³/µL	Elevated; possible reactive thrombocytosis
Prothrombin time (PT)	57 s	12 s	11-14 s	Significantly prolonged due to warfarin therapy
INR	4.5	–	2.0-3.0 (therapeutic)	Elevated; increased bleeding risk
Partial thromboplastin time (PTT)	30 s	30 s	25-35 s	Normal
Urinalysis and electrolytes	within normal limits	–	–	No abnormalities detected

**Fig. 4:**
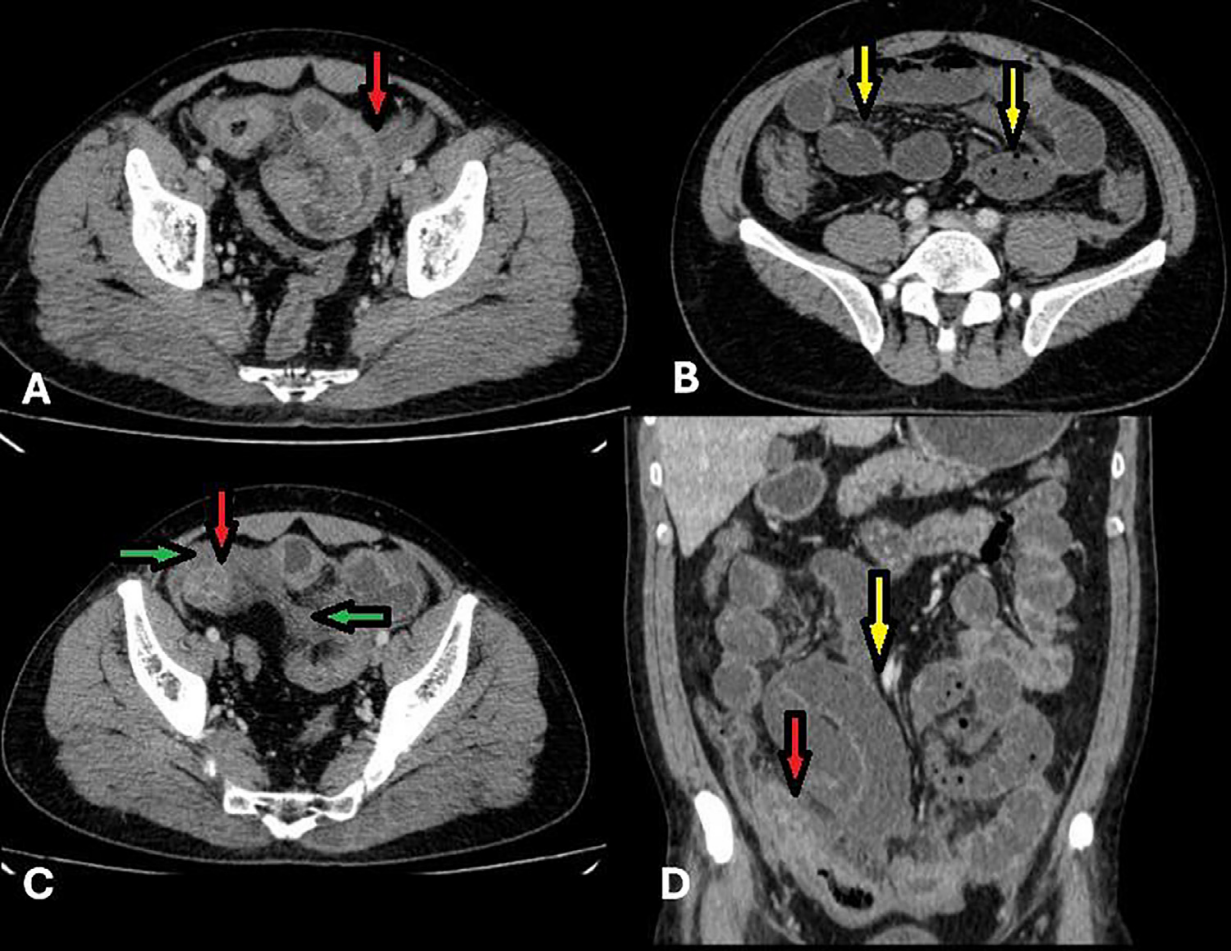
Abdominopelvic computed tomography (CT) with intravenous contrast injection (portal phase). (A) Circumferential long-segment symmetrical wall thickening is observed in the distal ileal loops, appearing hyperdense, suggestive of intramural hematoma (IMH) (red arrows). (B) More Proximal small bowel loops appear dilated, indicating partial obstruction (yellow arrows). Additionally, fluid is seen in mesentery. (C) Hyperdense free fluid is present in the para-colic gutters bilaterally, indicative of mild hemoperitoneum (green arrows). (D) Coronal view confirms circumferential long-segment thickening in the distal ileal loops (red arrows).

An abdominal ultrasound was performed due to a primary suspicion of acute appendicitis, which revealed bowel wall thickening in the terminal ileum, while the appendix appeared normal in size. A CT scan was subsequently recommended and performed. CT imaging with intravenous contrast was performed and demonstrated circumferential, long-segment symmetrical wall thickening appearing hyperdense in the distal ileal loops, indicative of an intramural hematoma. The affected ileal segment showed extensive bowel wall involvement without focal defects or perforation. Proximal to the lesion, dilated small bowel loops suggested partial obstruction caused by the mass effect of the hematoma. Hyperdense fluid was present at the root of the mesentery, consistent with mesenteric hemorrhage. Additionally, bilateral hyperdense free fluid in the paracolic gutters confirmed mild hemoperitoneum (Fig. 4). No signs of bowel ischemia or perforation were observed. The presence of mesenteric haziness and free fluid indicated a mild inflammatory response secondary to the hemorrhage. Given the absence of systemic signs of infection and based on imaging findings and the patient's history of anticoagulant use, a diagnosis of intramural hematoma was established.

The patient was managed conservatively with bowel rest, intravenous fluids, and supportive care. Given the markedly elevated INR, warfarin was discontinued, and anticoagulation was reversed with intravenous vitamin K and fresh frozen plasma. She was hospitalized for five days and demonstrated gradual clinical improvement. Once symptoms resolved, oral feeding was reintroduced. After multidisciplinary evaluation, she was discharged with instructions to initiate rivaroxaban 10 mg daily after a two-week interval. Although 20 mg daily is the standard therapeutic dose for atrial fibrillation, a reduced dose was chosen initially to minimize rebleeding risk in the context of recent gastrointestinal hemorrhage and moderate renal impairment. At the two-week follow-up, the patient remained asymptomatic, and repeat imaging confirmed complete resolution of the hematoma with normalization of bowel wall appearance. No complications or adverse events occurred during the hospitalization or follow-up period. The transition to rivaroxaban was uneventful, and the patient remained symptom-free.

## Discussion

SBIMH results from hemorrhage within the bowel wall, typically within the submucosa, leading to wall thickening and luminal narrowing. In anticoagulated patients, the pathophysiology involves the disruption of small blood vessels due to over-anticoagulation or coexisting conditions such as trauma or vascular fragility [3,8]. Over-anticoagulation therapy remains the most common etiology of spontaneous SBIMH in adults. Other risk factors include abdominal trauma, bleeding disorders (e.g., idiopathic thrombocytopenic purpura, hemophilia), malignancies (e.g., myeloma, leukemia, lymphoma, and pancreatic carcinoma), chemotherapy, pancreatitis, and vasculitis [9,10].

Among coagulation therapies, warfarin toxicity continues to be the predominant cause of SBIMH, whereas conventional heparin rarely induces this complication, likely due to stricter monitoring during inpatient care [11].

Intramural small-bowel hematoma is a rare but significant condition, with most cases occurring in patients undergoing long-term anticoagulation therapy. Common symptoms include abdominal pain (present in nearly all cases), nausea, vomiting, and gastrointestinal bleeding (reported in approximately 30% of cases) [10,12]. Obstructive symptoms resulting from luminal narrowing are frequently the mode of presentation, while massive hemorrhages are rare [9,13].

CT scan is considered the gold standard for diagnosing intramural hematomas. Characteristic CT findings include intramural hyperdensity (indicative of acute hemorrhage), circumferential bowel wall thickening, luminal narrowing, and occasionally, features of intestinal obstruction. The average length of hematomas has been reported at approximately 23 cm, with a minimum documented length of 8 cm. Measurement of hematoma length can help distinguish spontaneous SBIMH from malignant infiltration, such as metastasis or lymphoma. Notably, spontaneous hematomas typically involve longer bowel segments than traumatic hematomas. Although small bowel hematomas may occasionally extend into the colon, isolated intramural hematomas of the colon are exceedingly rare [10].

It is important to note that bowel wall thickening observed on CT imaging is not specific and may also be present in malignancy, inflammatory bowel disease, or ischemic conditions [14].

Acute abdominal pain, one of the most frequent complaints in the emergency department, requires a systematic and comprehensive approach. Studies by Macaluso and McNamara [1] and Cartwright and Knudson [2] emphasize the importance of considering rare etiologies, such as intramural hematoma, during evaluation to avoid delayed or missed diagnoses.

Spontaneous intramural small bowel hematoma secondary to anticoagulant therapy has been extensively documented in the literature. Abdel Samie and Theilmann [5] provided a comprehensive review of its pathogenesis, clinical manifestations, and management. Imaging, particularly CT, remains pivotal in the diagnostic process, as emphasized by Abbas et al. [6] and echoed in subsequent studies.

Anticoagulant therapy, particularly with warfarin, is a significant risk factor. Cases presented by Altikaya et al. and Uzun et al. [3,15] illustrate that even therapeutic levels of anticoagulation can result in SBIMH. These observations align with earlier findings by Goldfarb (1965) and Birns et al. [13], who first described coumarin-associated gastrointestinal complications.

Warfarin-induced SBIMH typically presents with symptoms of intestinal obstruction, such as abdominal pain, nausea, and vomiting. Abbas et al. [6] and Limmer and Clement [7] stressed the importance of distinguishing SBIMH from other causes of bowel obstruction to guide appropriate management strategies. Kang et al. [16] further supported the role of conservative management as the preferred initial approach, with favorable outcomes in most cases.

Although surgical intervention is rarely necessary, cases reported by Shaw et al. and Birla et al. [11] demonstrate that complications, such as bowel ischemia or perforation, may require operative management. Advances in diagnostic imaging and increased clinical awareness have significantly improved patient outcomes over time. Systematic reviews by Viniol et al. [4] and others highlight the necessity of a comprehensive diagnostic strategy, particularly for anticoagulated patients presenting with abdominal pain compared to previous reports, the two cases we presented offer unique insights:

• Higher INR levels: Both patients exhibited markedly elevated INR values (6.5 and 4.5), which are higher than typical ranges reported in similar case series, suggesting a strong correlation between excessive anticoagulation and the risk of SBIMH.
• Non-enhanced CT effectiveness: In the first case, the diagnosis was successfully achieved using a non-enhanced CT scan due to CKD limitations, demonstrating that non-contrast imaging can still effectively identify key signs of SBIMH when intravenous contrast administration is contraindicated.
• Transition to DOACs: Both cases involved successful transition from warfarin to direct oral anticoagulants (DOACs) after resolution of the hematoma, with no recurrence observed during follow-up. This supports the emerging evidence that DOACs may offer a safer alternative in patients at high risk for warfarin-related complications.

Although CT imaging in Case 1 clearly demonstrated features consistent with spontaneous intramural hematoma—such as long-segment hyperattenuating jejunal wall thickening without signs of perforation or free air—the surgical team elected to perform an exploratory laparotomy. This decision was based on the patient's persistent abdominal pain, diagnostic uncertainty, and concern for potential complications such as bowel ischemia, which could not be fully ruled out without direct visualization. Despite our radiologic impression and communication of the diagnosis, the surgeon proceeded with laparotomy and intraoperatively confirmed the presence of an intramural hematoma without any need for resection. The bowel was not compromised, and the abdomen was closed uneventfully. This case emphasizes the variability in clinical decision-making across disciplines and highlights the need for ongoing interdisciplinary collaboration and clinical judgment, especially in high-risk, anticoagulated patients .These aspects underline the importance of considering patient-specific factors, including renal function and anticoagulation management, when diagnosing and treating SBIMH

In conclusion, spontaneous small bowel intramural hematoma remains a diagnostic and therapeutic challenge. Clinicians should maintain a high index of suspicion for SBIMH in anticoagulated patients presenting with abdominal pain, particularly in cases of over-anticoagulation. Early CT imaging, even without contrast when necessary and timely conservative management are key to preventing severe complications. Furthermore, transitioning at-risk patients from warfarin to DOACs may reduce recurrence rates and improve long-term outcomes [15].

## Patient consent

Written informed consent was obtained from the patient for publication of this case report and any accompanying images. A copy of the written consent is available for review by the Editor-in-Chief of this journal.

## Appendix Supplementary materials

Image, application 1

## References

[1] Macaluso C.R., McNamara R.M. (2012). Evaluation and management of acute abdominal pain in the emergency department. Int J Gen Med.

[2] Cartwright S.L., Knudson M.P. (2008). Evaluation of acute abdominal pain in adults. Am Fam Physician.

[3] Viniol A., Keunecke C., Biroga T., Stadje R., Dornieden K., Bösner S. (2014). Studies of the symptom abdominal pain—a systematic review and meta-analysis. Fam Pract.

[4] Altikaya N., Parlakgümüş A., Demır Ş, Alkan Ö, Yildirim T. (2011). Small bowel obstruction caused by intramural hematoma secondary to warfarin therapy: a report of two cases. Turk J Gastroenterol.

[5] Abdel Samie A., Theilmann L. (2012). Detection and management of spontaneous intramural small bowel hematoma secondary to anticoagulant therapy. Expert Rev Gastroenterol Hepatol.

[6] Abbas M.A., Collins J.M., Olden K.W., Kelly K.A. (2002). Spontaneous intramural small-bowel hematoma: clinical presentation and long-term outcome. Arch Surg.

[7] Limmer A.M., Clement Z. (2017). Extensive small bowel intramural haematoma secondary to warfarin. J Surg Case Rep.

[8] Jones W.R., Hardin W.J., Davis J.T., Hardy J.D. (1971). Intramural hematoma of the duodenum: a review of the literature and case report. Ann Surg.

[9] Birla R.P., Mahawar K.K., Saw E.Y., Tabaqchali M.A., Woolfall P. (2008). Spontaneous intramural jejunal haematoma: a case report. Cases J.

[10] Abbas M.A., Collins J.M., Olden K.W. (2002). Spontaneous intramural small-bowel hematoma: imaging findings and outcome. AJR Am J Roentgenol.

[11] Shaw P.H., Ranganathan S., Gaines B. (2005). A spontaneous intramural hematoma of the bowel presenting as obstruction in a child receiving low-molecular-weight heparin. J Pediatr Hematol Oncol.

[12] Birns M.T., Katon R.M., Keller F. (1979). Intramural hematoma of the small intestine presenting with major upper gastrointestinal hemorrhage. Case report and review of the literature. Gastroenterology.

[13] Goldfarb W.B. (1965). Coumarin-induced intestinal obstruction. Ann Surg.

[14] Abdel Samie A., Sun R., Huber A., Höpfner W., Theilmann L. (2013). Spontaneous intramural small-bowel hematoma secondary to anticoagulant therapy: a case series. Med Klin Intensivmed Notfmed.

[15] Uzun M.A., Koksal N., Gunerhan Y., Sahin U.Y., Onur E., Ozkan O.F. (2007). Intestinal obstruction due to spontaneous intramural hematoma of the small intestine during warfarin use: a report of two cases. Eur J Emerg Med.

[16] Kang E.A., Han S.J., Chun J., Lee H.J., Chung H., Im J.P. (2019). Clinical features and outcomes in spontaneous intramural small bowel hematoma: cohort study and literature review. Intest Res.

