# Supplementary material for: Spontaneous intramural hematoma of the small bowel in anticoagulated patients—Insights into management: A case report
Source: Radiol Case Rep. 2025 Jul 28;20(10):5189–95. doi: 10.1016/j.radcr.2025.06.079 (PMC12320024; doi:10.1016/j.radcr.2025.06.079)
Supplement: Supplementary file 1 [file mmc1.docx]

# **Spontaneous Intramural Hematoma of the Small Bowel in Anticoagulated Patients: Two Case Reports**

## ****Keywords****

Intramural hematoma, Small bowel, Anticoagulation therapy, Warfarin complications, Abdominal pain, Computed tomography (CT), Direct oral anticoagulants (DOACs), Case report

## ****Abstract****

### **Introduction**

Intramural hematoma of the small bowel (SBIMH) is a rare but clinically significant complication of anticoagulation therapy, particularly in patients receiving warfarin. Delayed diagnosis can lead to life-threatening complications, including bowel obstruction and perforation.

### **Patient Information and Clinical Findings**

**Case 1**: A 72-year-old male on long-term warfarin therapy for heart valve replacement presented with severe abdominal pain, nausea, and vomiting. His INR was markedly elevated at 6.5. CT imaging revealed a jejunal intramural hematoma.
**Case 2**: A 51-year-old female on warfarin for atrial fibrillation presented with acute abdominal pain and partial bowel obstruction. Her INR was 4.5, and CT demonstrated an ileal intramural hematoma with associated hemoperitoneum.

### **Diagnosis, Interventions, and Outcomes**

Both cases were diagnosed via CT imaging. Management involved discontinuing warfarin, administering vitamin K and fresh-frozen plasma, and providing supportive care. Case 1 required surgical exploration, while Case 2 was managed conservatively. Both patients recovered fully and were transitioned to DOACs without recurrence.

### **Conclusion**

Early diagnosis and multidisciplinary management of SBIMH in anticoagulated patients are essential for favorable outcomes. Transitioning from warfarin to DOACs may lower recurrence risk. Clinicians should maintain a high index of suspicion for SBIMH in anticoagulated patients presenting with abdominal pain.

## ****Introduction****

Abdominal pain is a common presentation in emergency settings, often requiring careful diagnostic assessment. Small bowel intramural hematoma (SBIMH) is a rare but potentially severe complication of anticoagulation therapy, primarily associated with warfarin. It occurs in approximately 1 in 2500 anticoagulated patients annually. Though most cases can be managed conservatively, timely diagnosis is critical to preventing complications such as obstruction, perforation, or peritonitis. This report presents two cases of SBIMH in anticoagulated patients, highlighting the role of early imaging and anticoagulation management in optimizing patient outcomes.

## ****Patient Information****

### **Case 1**

**Patient Demographics**: 72-year-old male, retired accountant, BMI: 28.5 kg/m²
**Primary Concerns**: Gradual onset of abdominal pain (localized to periumbilical and left lower quadrant), nausea, vomiting
**Medical History**: Heart valve replacement (warfarin 5 mg daily for 5 years), hypertension (Amlodipine 5 mg daily), stage III chronic kidney disease
**Family History**: No significant bleeding disorders or gastrointestinal diseases
**Social History**: Former smoker (25-pack-year history, quit 5 years ago)
**Past Interventions**: None relevant

### **Case 2**

**Patient Demographics**: 51-year-old female, retired teacher, BMI: 26.3 kg/m²
**Primary Concerns**: Acute, severe abdominal pain (right lower quadrant), mild bloating, constipation
**Medical History**: Atrial fibrillation (warfarin 5 mg daily for 8 months), type 2 diabetes (Metformin 500 mg twice daily), osteoarthritis (occasional NSAIDs)
**Family History**: No significant bleeding disorders or gastrointestinal diseases
**Social History**: Non-smoker
**Past Interventions**: None relevant

## ****Clinical Findings****

### **Case 1**

- **Vital Signs**: BP: 135/80 mmHg, HR: 80 bpm, RR: 16/min, afebrile
- **Physical Exam**: Mild periumbilical tenderness, hypoactive bowel sounds, no guarding or rebound tenderness
- **Laboratory Findings**:
  - INR: **6.5** (therapeutic range: 2-3)
  - PT: 79 seconds (prolonged)
  - Hemoglobin: 12 g/dL (mild anemia)
  - Creatinine: 2.7 mg/dL (elevated due to CKD)

### **Case 2**

- **Vital Signs**: BP: 125/78 mmHg, HR: 72 bpm (irregular), RR: 16/min, afebrile
- **Physical Exam**: Right lower quadrant tenderness, mild bruising on extremities, hypoactive bowel sounds
- **Laboratory Findings**:
  - INR: **4.5**
  - PT: 57 seconds (prolonged)
  - Hemoglobin: 13 g/dL
  - Platelets: 386 × 10³/µL (elevated, reactive thrombocytosis)

## ****Timeline****

| **Event** | **Case 1** | **Case 2** |
| --- | --- | --- |
| Symptom onset | 48 hours prior | 12 hours prior |
| ER presentation | Abdominal pain, vomiting | Abdominal pain, constipation |
| INR levels | 6.5 | 4.5 |
| Imaging | CT scan: Jejunal IMH | CT scan: Ileal IMH + hemoperitoneum |
| Intervention | Warfarin reversal, surgery | Warfarin reversal, conservative management |
| Follow-up | No recurrence at 3 weeks | No recurrence at 2 weeks |

## ****Diagnostic Assessment****

- **Case 1**: Non-contrast CT revealed a hyperattenuated long-segment circumferential wall thickening in the jejunum, consistent with intramural hemorrhage.
- **Case 2**: CT imaging showed hyperdense circumferential wall thickening of the distal ileum, mesenteric hemorrhage, and mild hemoperitoneum.

### **Differential Diagnoses Considered**

1. Mechanical bowel obstruction
2. Gastrointestinal perforation
3. Ischemic bowel disease

## ****Therapeutic Intervention****

### **Case 1**

- Warfarin discontinued
- Fresh-frozen plasma and vitamin K administered
- **Surgical intervention**: Laparotomy confirmed localized intramural hemorrhage without active bleeding → No resection required
- **Outcome**: Complete resolution in 3 weeks

### **Case 2**

- Warfarin discontinued
- Supportive care: Bowel rest, IV fluids
- **Conservative management**: No surgical intervention required
- **Outcome**: Full recovery in 2 weeks

## ****Follow-up and Outcomes****

- **Both patients transitioned from warfarin to DOACs**
- **No recurrence at follow-up**
- **No adverse events or complications**

## ****Discussion****

SBIMH is a rare but critical diagnosis in anticoagulated patients presenting with abdominal pain. **CT imaging remains the gold standard** for diagnosis, revealing hyperdensity and bowel wall thickening. **Conservative management is effective in most cases**, with surgery reserved for complications. Transitioning from warfarin to **DOACs appears to be a safer alternative** to minimize recurrence.

### **Strengths and Limitations**

- **Strengths**: Early imaging facilitated timely intervention and conservative management.
- **Limitations**: Reliance on clinical suspicion; delayed imaging in hemodynamically unstable patients may hinder diagnosis.

### **Primary Takeaway Lessons**

1. **Non-enhanced CT is critical for diagnosing SBIMH in anticoagulated patients.**
2. **Narrowing CT window width enhances visualization of intramural hyperdensity.**
3. **Early recognition enables conservative management, reducing the need for surgery.**
4. **Transitioning to DOACs may reduce recurrence risk.**

## ****Patient Perspective****

Both patients reported relief after treatment. Case 1 expressed initial concern about surgical intervention but was reassured by the outcome. Case 2 appreciated the transition to DOACs, avoiding frequent INR monitoring.

## ****Informed Consent****

Informed consent was obtained from both patients for the publication of their cases.
